# Supplementary material for: Ancient and current association of the Trichinella complex with negative-sense and double-stranded RNA viruses
Source: J Virol. 2026 Jul 28;100(8):e00485-26. doi: 10.1128/jvi.00485-26 (PMC13483474; doi:10.1128/jvi.00485-26)
Supplement: Supplementary file 1 [file jvi.00485-26-s0001.pdf]

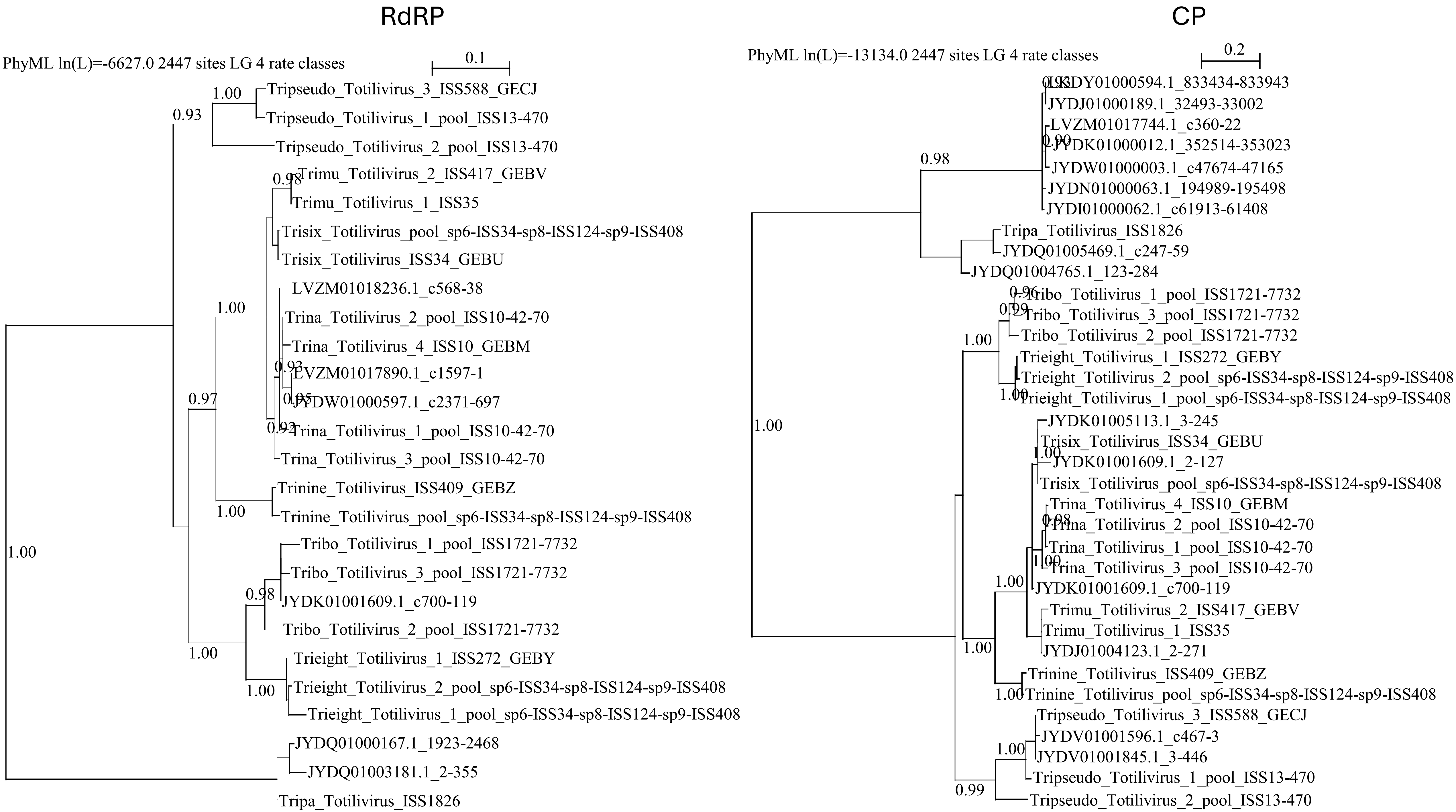

Supp Fig 1: Comparison of the phylogenetic relationship among proteins encoded by EVE and Totiliviruses identified in different *Trichinella* species. Phylogenetic tree of the RNA-directed RNA polymerase (RdRP) and Capsid (CP) proteins. The trees were inferred in PhyML using the LG substitution model. Branch points indicate that results of Shimodaira-Hasgawa branch test > 0.9.
